# Optic compressive neuropathy secondary to anterior clinoid mucocele: diagnostic approach, a case report

**DOI:** 10.1093/jscr/rjac362

**Published:** 2022-09-06

**Authors:** Francisco J Medina-Valencia, Enrique C García-Pretelt, Verónica Alzate-Carvajal, Camilo E Moreno-Huertas, Isabella Moreno-Arango

**Affiliations:** Departamento de Neurorradiología, Fundación Valle del Lili, Cali, Colombia; Departamento de Radiología e Imágenes Diagnósticas, Facultad de Medicina, Universidad Icesi, Cali, Colombia; Departamento de Neurocirugía, Facultad de Medicina, Universidad Icesi, Cali, Colombia; Departamento de Neurocirugía, Fundación Valle del Lili, Cali, Colombia; Centro de Investigaciones Clínicas, Fundación Valle del Lili, Cali, Colombia

**Keywords:** case report, anterior clinoid process, mucocele, optic neuropathy, blindness

## Abstract

Anterior clinoid process (ACP) mucoceles are an uncommon entity and an even rarer cause of visual impairment. We review the case of a 62-year-old female with a 2-year history of progressive right-sided monocular vision loss. Paranasal sinus computed tomography (CT) scan showed bilateral ACP pneumatization. A soft tissue density mass occupied the right ACP with bone expansion and compression of the right orbital canal. An endonasal approach was performed with total vision recovery. Dedicated images are necessary to diagnose ACP. On CT, the affected sinus will have bone erosions. On magnetic resonance imaging, the signal intensity is determined by its protein concentration and mobile water protons. ACP mucoceles’ accurate diagnosis determinates the treatment and surgical approach. Finally, the correct management selection will determine the retrieval of the visual ability.

## INTRODUCTION

The anterior clinoid process (ACP) is a bony projection on the medial aspect of the posterior border of the lesser sphenoid wing. Its pneumatization is not a rare incidental finding, with a reported incidence of 4–29.3% [1]. Its occurrence carries an increased risk of cerebrospinal fluid leakage after skull base surgery, especially after anterior clinoidectomy.

Blockage of the drainage of a paranasal sinus may occur usually because of repeated inflammation or, in some rare cases, as a congenital variation [2]. It may lead to the accumulation of secretions, resulting in a mucocele [3], with subsequent mass effect and bony remodeling. Sphenoid mucoceles account for 1% of all paranasal sinus mucoceles, and involvement of the ACP has been sparsely reported in the past [4].

Due to its proximity to the optic nerve, ACP mucoceles may, although very rare, be a cause of orbital apex syndrome—compressive optic neuropathy [5]. Computed tomography (CT) and magnetic resonance imaging (MRI) play an essential role in characterizing the lesion and its extension. We report a case according to the Surgical CAse REport (SCARE) criteria [6].

## PRESENTATION OF CASE

A 62-year-old woman with no medical background presented to the emergency department with a 2-year history of progressive right-sided monocular vision loss. The ophthalmological assessment evidenced a visual acuity of 20/200 −1 PH 20/100 + 2 in her right eye, relative left afferent pupillary defect and pale temporal neuroretinal ring with normal bilateral extraocular movements and normal vision of the left eye.

**Figure 1:**
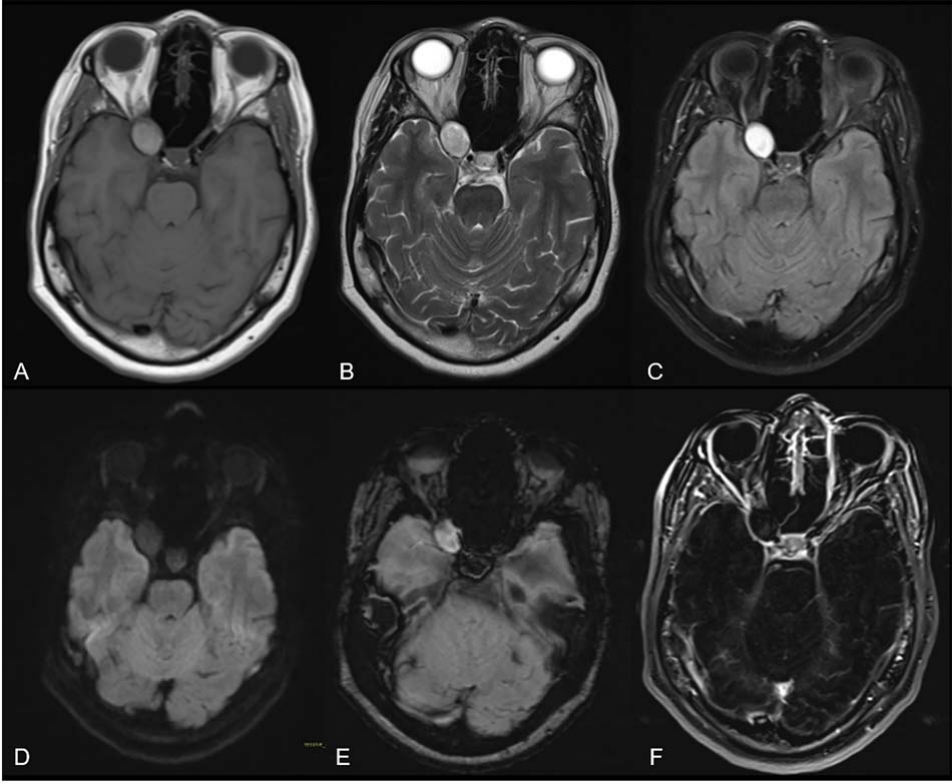
Axial cuts of brain MRI. T1W (**A**), T2W (**B**), FLAIR (**C**), DWI (**D**), SWI (**E**) and contrast-enhanced T1W images with subtraction (**F**); a rounded mass in the right ACP with intrinsic T1 high-intensity signal, suggesting a proteinaceous content with smooth, linear and peripheral mucosal enhancement; no restricted diffusion or susceptibility artifacts were observed.

At arrival, under the suspicion of a supraclinoid internal carotid aneurysm, an MRI angiography was performed, evidencing a rounded proteinaceous mass in the right ACP with compression of the optic nerve suggestive of an ACP mucocele (Fig. 1). No vascular abnormalities were demonstrated. A conventional subtraction angiography was also performed with no aneurysms detected.

**Figure 2:**
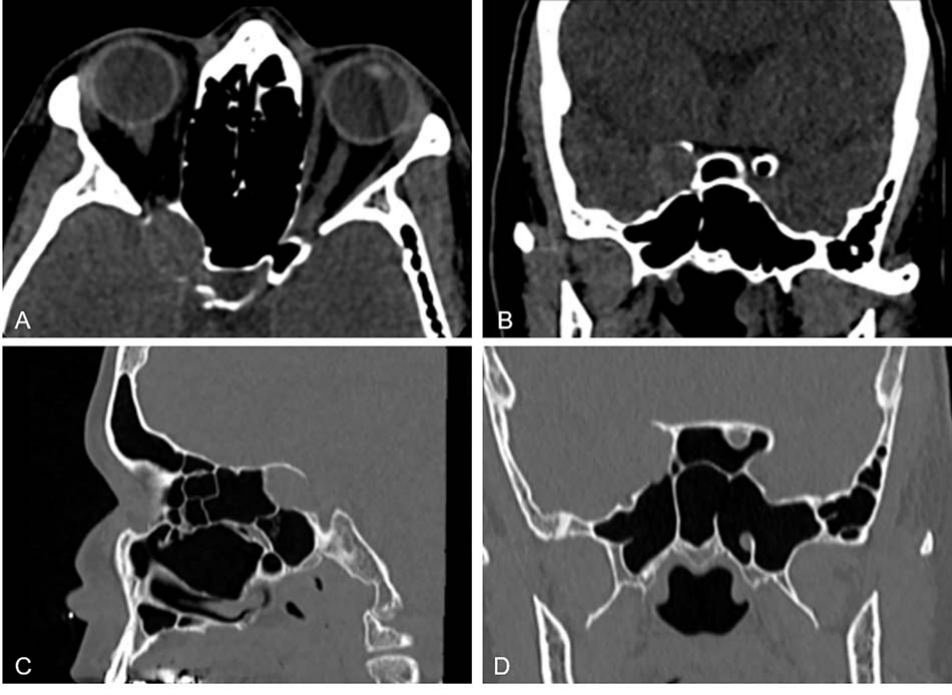
Paranasal sinus CT scans multiplanar reconstructions in axial (**A**), coronal (**B**, **D**) and sagittal (**C**) planes, with modified window (A and B) and bone algorithm (C and D); they show the right ACP with thin eroded walls, occupied by an oblong, soft tissue density mass that expands the bone cortical and compresses the right orbital apex.

A pre-surgical paranasal sinus CT scan showed bilateral pneumatization of ACP; the right one had thin eroded bony walls and was occupied by a soft tissue density lesion that compressed the orbital apex (Fig. 2).

**Figure 3:**
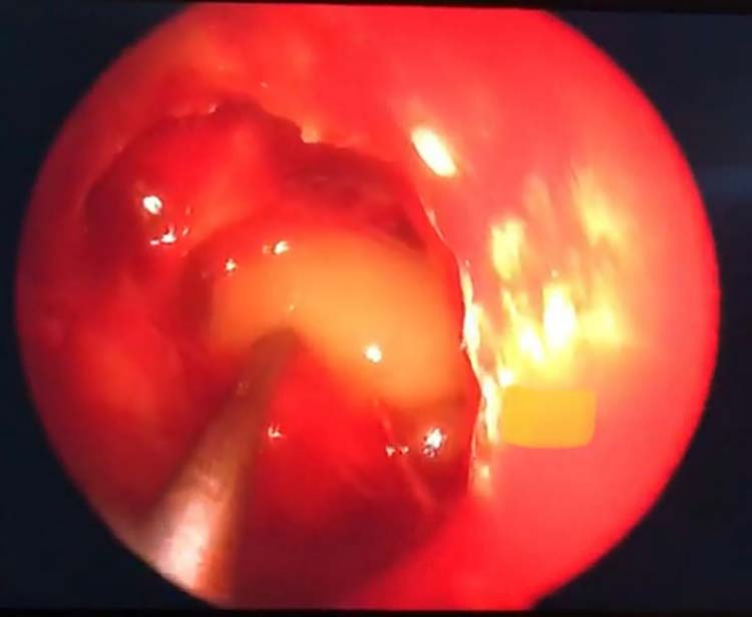
Grayish soft tissue lesion occupying the right ACP consistent with mucocele.

An endoscopic endonasal approach with neuronavigation through an ethmoidal approach was performed 8 days after arrival by an experienced neurosurgeon. The expanding ACP was drilled, allowing the exposure of a soft, grayish lesion consistent with mucocele (Fig. 3), which was resected and sent to pathology. A wide osteotomy of the posterior ethmoidal cell and ACP was done to allow the optic nerve decompression.

The post-operative course was uncomplicated, with the right eye visual acuity of 20/20 2 months after surgery. Follow-up CT showed a complete resolution of the anterior clinoid mucocele with no residual lesion.

## DISCUSSION

ACP mucoceles are an uncommon entity and a rare cause of visual impairment. Dedicated images are necessary to diagnose and treat them. On CT, the affected sinus will have rounded, eroded and thinned walls [7]. The signal intensity of sinonasal secretions of mucoceles on MRI is based on its protein concentration and mobile water protons. As protein concentration increases, the signal intensity on T1WI of sinus secretions changes from hypointense to hyperintense and to hypointense again. On T2WI, mucoceles are initially hyperintense, but the signal intensity decreases as the protein concentration and viscosity increase [8]. Chronically obstructed sinus secretions may have any combination of signal intensities.

The treatment of paranasal sinus mucocele aims to relieve the compressive effect on the surrounding structures and to prevent recurrences. Choosing the most appropriate surgical approach allows complete resection and minimal invasion with the least possible complications.

Pterional and subtemporal approaches were classically used to achieve total decompression and resection. However, there was a high rate of complications and morbidity. Using an endoscopic endonasal approach with a microsurgical technique has shown good anatomic and functional results [9, 10], and in some cases, with complete recovery of visual loss following endoscopic resection [11].

In this case, head images allowed an accurate diagnosis of a lesion in the right ACP with optical nerve compression, which was treated using a mini-invasive technique through an endoscopic trans-ethmoidal approach, with a total recovery of the visual deficit.
